# Diagnostic Value of Muscle Biopsy for the Evaluation of Adult Myopathy in Daily Clinical Practice

**DOI:** 10.3390/diagnostics15243102

**Published:** 2025-12-06

**Authors:** Vera E. A. Kleinveld, Julia Wanschitz, Anna Hotter, Johannes A. Mayr, Romana Höftberger, Wolfgang N. Löscher, Corinne G. C. Horlings

**Affiliations:** 1 Department of Neurology, Medical University of Innsbruck, 6020 Innsbruck, Austriawolfgang.loescher@tirol-kliniken.at (W.N.L.); 2 University Children’s Hospital, Salzburger Landeskliniken (SALK), Paracelsus Medical University, 5020 Salzburg, Austria; 3 Division of Neuropathology and Neurochemistry, Department of Neurology, Medical University of Vienna, 1090 Vienna, Austria

**Keywords:** muscle biopsy, diagnosis, myopathy, histopathology, myositis

## Abstract

**Background/Objectives:** Muscle biopsy is traditionally considered a cornerstone in the diagnosis of myopathies. Advances in the clinical and laboratory evaluation of myopathies warrant re-evaluation of the diagnostic yield. **Methods:** Results of muscle biopsies performed between 1 January 2009 and 31 January 2023 in patients with symptoms indicative of myopathy were evaluated and set in relation to clinical diagnosis, based on phenotype, electromyography, laboratory results, and available antibody testing. Biopsies were classified as diagnostic (changed or specified clinical diagnosis), confirmative (same as clinical diagnosis), or non-informative (normal/unspecific findings). Genetic testing followed muscle biopsy at later follow-up, upon availability of genetic testing. **Results:** One-hundred sixty-two patients were included and divided into five groups based on clinical phenotype: inflammatory myopathy, *n* = 54; mitochondrial myopathy, *n* = 33; muscular dystrophy, *n* = 23; metabolic myopathy, *n* = 3; and non-specific phenotype (isolated hyperCKemia/myalgia), *n* = 49. Muscle biopsy was diagnostic in 21.0%, confirmative in 38.3% and non-informative in 40.7% of patients. The percentage of diagnostic biopsies was 66.7% in metabolic myopathy, 54.5% in mitochondrial myopathy, 17.4% in muscular dystrophy, 14.8% in inflammatory myopathy, and 4.1% in the non-specific phenotype. **Conclusions:** Overall, in our cohort, muscle biopsy yielded a new diagnosis or additional information in 21.0% of patients. In the majority, a diagnosis was established based on clinical and laboratory evaluation, and muscle biopsy was either confirmative or non-informative. We propose muscle biopsy in cases where serological and genetic tests are inconclusive, in the presence of specific signs indicative of myopathy, or when in-tissue genetic testing is necessary to obtain a comprehensive diagnosis.

## 1. Introduction

Myopathies are a heterogeneous group of disorders affecting the structure, metabolism or channel functions of skeletal muscle. They may present with muscle weakness, atrophy, exercise intolerance, stiffness, abnormal electromyography (EMG) findings, or elevated creatine kinase (CK) levels without sensory symptoms [1,2,3,4]. Myopathies can either be inherited (e.g., muscular dystrophies, mitochondrial myopathies, and other metabolic myopathies) or acquired (e.g., immune-mediated and toxic myopathies) and muscle biopsy assessment profoundly contributed to the characterization of the underlying pathogenic processes. With the application of a wide range of techniques on frozen tissue, electron microscopy muscle biopsy represented the main tool for diagnosis for many decades [5].

The diagnostic evaluation of myopathies, however, has changed over the years with advanced genetics and the detection of myositis-specific antibodies [6]. Despite these developments, not all cases of myopathy can be solved by blood testing. In only approximately 20% of inflammatory myopathies, a known antibody can be found [6] and a pathogenic genetic variant is found in 11–62% of patients with limb girdle muscular weakness [7,8,9]. In addition, cases with clinically unclear presentations exist. For these cases, muscle biopsies may still be a useful tool in the diagnostic work-up.

For adult populations with suspected, clinically undefined, myopathy based on weakness and/or hyperCKemia, the overall diagnostic yield ranges from 36% [10] to 43% [11]. However, there is a lack of real-world studies assessing the diagnostic yield of muscle biopsy when stratified by patient phenotype indicative of specific neuromuscular disorders.

For this reason, this study aims to define the diagnostic yield of muscle biopsies in several clinically well-defined myopathy phenotypes. The results of this study are intended to determine the most rational indications for muscle biopsies in clinical practice and to serve as a guide in the decision-making process for whether a muscle biopsy should be performed.

## 2. Materials and Methods

In this retrospective study, clinical and pathological data from all consecutive adult patients who underwent muscle biopsies in the diagnostic work-up of myopathy at the Department of Neurology, Medical University of Innsbruck (Austria) between 1 January 2009 and 31 January 2023 were reviewed. Clinical and demographical data were retrieved from electronic patient records. When multiple CK values were available, the highest value before muscle biopsy was collected. HyperCKemia was defined as 1.5 times the upper limit of normal according to EFNS guidelines [12]. Local laboratory reference values are sex-specific. As part of routine diagnostic follow-up, nerve conduction studies (NCS) and electromyography (EMG) were performed. Patients with suspected muscular dystrophy were only biopsied when genetic panels or dried blood spot tests for Pompe disease were not yet available or when genetic testing was negative. Imaging was not included in the routine diagnostic work-up.

All muscle biopsies were performed by an open procedure in muscles that were clinically affected and had a Medical Research Council (MRC) grade higher than 3, to obtain tissue that revealed pathological disease characteristics but not end-stage morphology [13]. This was mostly the lateral vastus, deltoid, or biceps brachii muscle, and the sample was 0.5–1 cm^3^ after being trimmed of fat, coagulated blood, and connective tissue. For all muscle biopsies, the greater part was immediately snap-frozen in liquid nitrogen and stored at −80 °C for standard histology assessment, enzyme-histochemical and immunohistochemical assessment, biochemical analysis, Western blot, and DNA extraction. The other part of the muscle biopsy was fixed in 4% glutaraldehyde for electron microscopic examination. There were no major changes to the analyses conducted during the study period. The maximum period of time between the muscle biopsy procedure and the time until analysis was 4 weeks.

The routine muscle biopsy staining protocol included the following stains: hematoxylin and eosin (H&E), Gomori-Trichrome, NADH, SDH, COX, PAS, Oil-red-O, and ATP-ase at pH 9.2, as well as immunostains for LCA, CD68, and major histocompatibility complex (MHC) class I. The protocol was reviewed by one of the authors (JW). When initial staining revealed abnormalities, extensive immunohistochemistry and electron microscopy were performed at the Division of Neuropathology and Neurochemistry, Medical University of Vienna, Austria. When abnormal numbers and/or morphological alterations of mitochondria were detected, cryo-conserved tissue was sent to the University Hospital of Salzburg, Austria for biochemical analysis of respiratory chain enzymes and analysis of mitochondrial DNA. A detailed overview of diagnostic procedures on muscle biopsies is provided in Supplementary Table S3.

Based on the presence or absence of pathognomonic findings, muscle biopsies were categorized as follows.

### 2.1. Inflammatory Myopathy (IIM)

The composition and distribution of inflammatory infiltrates, the presence of perifascicular atrophy and capillary depletion, perimysial fragmentation, myo-necrosis, regeneration, CD8 T-cellular invasion of non-necrotic muscle fibers, the pattern of MHC class I upregulation and deposition of C5b-9, and the presence of rimmed vacuoles and/or cytoplasmic protein aggregates were used for the histological subcategorization of IIMs into Dermatomyositis (DM), Antisynthetase Syndrome/Overlap Myositis (AsyS/OM), Polymyositis (PM; delineation from Inclusion Body Myositis by the absence of rimmed vacuoles, cytoplasmic protein aggregates, and mitochondrial abnormalities), Inclusion Body Myositis (IBM), Immune-Mediated Necrotizing Myopathy (IMNM), or unspecific inflammatory changes according to international recommendations [14,15,16].

### 2.2. Muscular Dystrophy

Biopsies with the presence of features indicating muscular dystrophy, e.g., prominent fiber caliber variation, internalized nuclei, fiber splitting, whorled fibers, myo-necrosis, regeneration, endomysial fibrosis, and fatty infiltration, were further classified according to specific abnormal protein expression or deficiency (specified in Supplementary Table S3).

### 2.3. Metabolic Myopathy

For the diagnosis of metabolic myopathy, in case of abnormal accumulation of glycogen or lipids upon initial analysis, tissue was analyzed for metabolic enzymes involved in glycogen regulation or lipid metabolism.

### 2.4. Mitochondrial Myopathy

The presence of ragged red fibers on Gomori-Trichrome stain, hyperreactive fibers/negative fibers on SHD/COX stains, and the presence of abnormal mitochondria using electron microscopy warranted further analysis of respiratory chain enzymes and mitochondrial DNA on muscle tissue.

### 2.5. Non-Specific/Normal

Biopsies were classified as non-specific by the presence of non-pathognomonic myopathic changes (e.g., type I fiber predominance, type II fiber atrophy, moth-eaten fibers) that were not in concordance with the above categories or normal findings.

The clinical diagnosis before muscle biopsy was extracted from digital patient records and was based on the clinical phenotype, muscle enzymes, available antibody testing in serum, and electrophysiological studies, e.g., NCS and EMG. The clinical diagnosis was established by the treating clinician with the agreement of two or more neurologists specializing in neuromuscular disorders and electrophysiology, including an experienced myopathologist. For inflammatory myopathy, the clinical diagnosis was established in accordance with the 2017 EULAR/ACR IIM classification criteria without muscle biopsy [17]. The diagnosis was further specified using serum antibody testing for MSAs known at the time of biopsy, including anti-SRP, anti-TIF-1 gamma, anti-NPX, anti-SAE, anti-Jo, and other antisynthetase antibodies using immunoblots (EUROIMMUN AG, Lübeck, Germany), as well as MAAs such as anti-Ro52, anti PM-Scl, and anti-Ku (determined at the local rheumatology lab). Anti-HMGCoA and anti-MUP 44 antibodies were analyzed in external labs (MVZ Laboratory, Karlsruhe, Germany and Labor Stöcker, Groß Grönau, Germany). For other forms of myopathy, classification was based on patient presentation and electrophysiological studies. Generally, patients with a clinical diagnosis of muscular dystrophy presented with progressive proximal muscle weakness and additional features, such as calf hypertrophy, scapular winging, hyperlordosis, and rigidity. Patients with a clinical diagnosis of metabolic myopathy presented with reversible exercise intolerance and recurrent attacks of myalgia or myoglobinuria, with or without proximal weakness and/or second-wind or out-of-wind phenomena. Patients with mitochondrial myopathy presented with progressive external ophthalmoplegia (PEO) and/or weakness and exercise intolerance. Patients with only myalgia, with or without hyperCKemia, and lacking pathognomic signs and symptoms of a myopathy, were classified as having a non-specific phenotype.

The diagnostic value of the biopsy was categorize as diagnostic, confirmative, or non-informative. Diagnostic biopsies provided a new diagnosis or etiological subclassification; confirmative biopsies confirmed the clinical diagnosis and provided no new information; non-informative biopsies showed non-specific or normal findings.

### 2.6. Statistical Methods

Shapiro–Wilk tests were performed to test the normality of the data. We used Kruskal–Wallis tests to compare non-normally distributed variables among multiple groups and Mann–Whitney U test to compare non-normally distributed variables between two groups. Correlation analysis was performed using Spearman’s rank for non-parametrical variables. To assess the overlap between the clinical diagnosis and the biopsy diagnosis, concordance rates were calculated. A two-tailed *p* < 0.05 was considered statistically significant. Results are presented as the mean and standard deviation if not stated otherwise. All statistical analyses were performed using IBM^®^ SPSS (version 28.0.1.0 (142)), and alluvial plots were generated using Flourish Studio (Canva UK Operations Ltd., London, UK). Ethics committee approval was waived due to the retrospective nature of this study. This study was performed in accordance with the Declaration of Helsinki.

## 3. Results

All 162 patients who underwent a muscle biopsy at our institute between 1 January 2009 and 31 January 2023 were included. Clinical and demographic data are summarized in Table 1, separated per clinical phenotype (established before biopsy). The CK-value of patients with diagnostic and confirmative biopsies (mean of 4958.1 ± 24,964.6 IU/L) was significantly higher than that of patients with non-informative biopsies (mean of 939.8 ± 2117.0 IU/L, *U* = 3918.500, *p* < 0.01).

**Figure 1:**
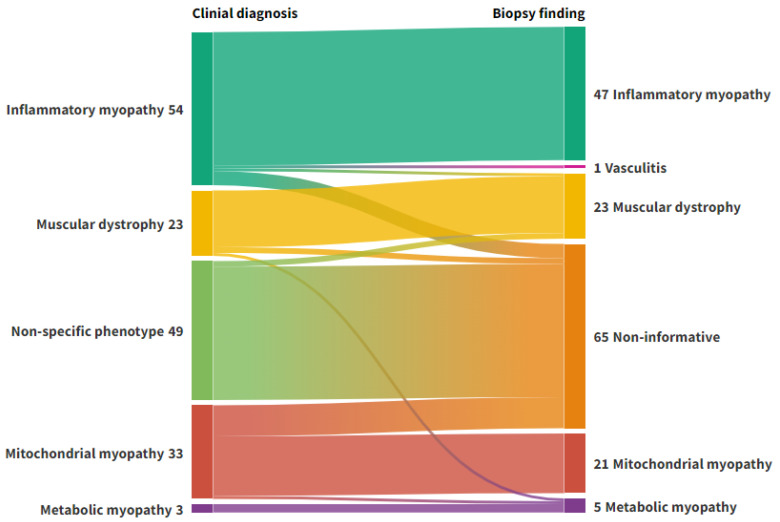
Alluvial plot representing the flow of diagnoses from clinical phenotyping to muscle biopsy. Left axis: Clinical diagnosis. Right axis: Biopsy diagnosis. Non-informative: Normal biopsies or biopsies with discrete unspecific changes. Figure created with Flourish Studio.

After biopsy, 95 patients had a final diagnosis of a myopathy, and 1 patient with clinical suspicion of myopathy had signs of vasculitis on biopsy, which was considered diagnostic. Sixty-six biopsies were normal or non-specific. Clinical and biopsy diagnoses are shown in Figure 1. The diagnostic yield of the biopsies, per clinical diagnosis, are listed in Table 2. For patients assigned a subcategory in both the clinical diagnosis (e.g., DM in the IIM group) and in the biopsy diagnosis (*n* = 51), the concordance rate was 92.2%.

### 3.1. Inflammatory Myopathy

Among those in whom inflammatory myopathy was clinically suspected (*n* = 54), MSA testing was performed in 41 patients: 25 (61.0%) patients tested positive, and 16 (39.0%) patients tested negative. Antibody profiles are provided in Table 3. In patients without serum antibody testing, eight biopsies were confirmative, two biopsies were diagnostic, while three were non-informative.

The diagnostic biopsies provided a new diagnosis (*n* = 1 muscular dystrophy, *n* = 1 vasculitis) or refined the diagnosis (*n* = 4 immune-mediated necrotizing myopathy (IMNM), *n* = 1 overlap syndrome, *n* = 1 Polymyositis (PM)). Within the group of seropositive patients (*n* = 25), 21 biopsies were confirmative, 2 were diagnostic, while 2 were non-informative. For the group of seronegative patients (*n* = 16), 12 biopsies were confirmative, while 4 were diagnostic.

**Figure 2:**
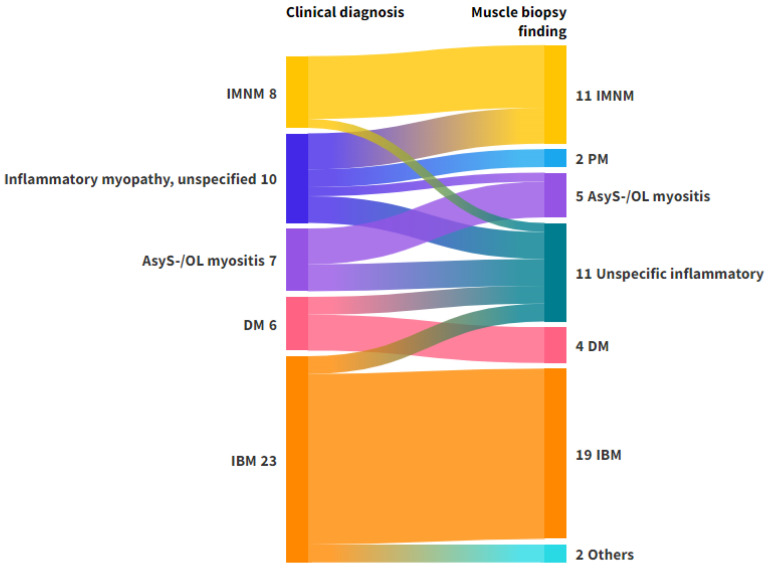
Alluvial plot representing the flow of diagnoses from clinical phenotyping to muscle biopsy in patients with a clinical diagnosis of inflammatory myopathy. Left axis: Clinical diagnosis. Right axis: Biopsy diagnosis. The clinical diagnosis ‘Inflammatory myopathy, unspecified’ includes patients with suspected myositis who could not be classified on clinical grounds, including antibody testing. The muscle biopsy finding ‘others’ consisted of patients with vasculitis (*n* = 1) and muscular dystrophy (*n* = 1). The muscle biopsy finding ‘unspecific inflammatory’ was used for biopsies with inflammatory findings, which were not specific for any of the subtypes. Abbreviations: AsyS-/OL myositis, antisynthetase syndrome/overlap myositis; DM, dermatomyositis; IBM, Inclusion Body Myositis; IMNM, immune-mediated necrotizing myositis; PM, Polymyositis. Figure created with Flourish Studio.

A total of 90.7% of the patients received a subclassification in the clinical diagnosis, and the concordance rate to the biopsy diagnosis was 95.7%. For IBM, the largest group within the category of inflammatory myopathies, the concordance rate was 95.2%. A comprehensive overview of the clinical diagnoses and muscle biopsy findings can be found in Figure 2.

### 3.2. Mitochondrial Myopathy

When the clinical diagnosis was mitochondrial myopathy (*n* = 33), 18 biopsies were diagnostic, 4 confirmative, and 11 non-informative. In one patient, the diagnosis was changed to secondary toxic metabolic myopathy based on biopsy findings. In the other 17 biopsies, genetic testing on biopsy tissue revealed a mutation or deletion of mitochondrial DNA. In those genetically tested using biopsy tissue, 4/21 biopsies revealed negative results.

### 3.3. Muscular Dystrophy

In patients with a clinical diagnosis of muscular dystrophy (*n* = 23), 4 biopsies were diagnostic, and 16 were confirmative. For diagnostic biopsies, one biopsy showed morphological signs of glycogen storage disease and an alfa-1,4-glucosidase deficiency (confirmed with a mutation in the *GAA* gene). For three biopsies, a subtype diagnosis could be made based on the immunoblot results (see Table S1 in the Supplementary Material). Patients with a clinical suspicion of muscular dystrophy were only biopsied in the period before multi-gene panel tests became commercially available. During later follow-up, upon increased availability of multi-gene panel testing, genetic testing was performed in 19 patients, and in 12 of those, genetic testing revealed a mutation (*n* = 10 confirmative biopsies, *n* = 2 non-informative biopsies).

### 3.4. Metabolic Myopathy

In suspected metabolic myopathy (*n* = 3), two biopsies were diagnostic by providing a subcategory (lipid storage myopathy). Genetic testing was performed in one patient after biopsy, and a carnitine palmitoyltransferase (CPT2) deficiency was genetically confirmed.

### 3.5. Non -Specific Phenotype

For the group of patients with a non-specific phenotype (*n* = 49), two biopsies were diagnostic, showing dystrophic changes (see Table S2 in the Supplements), while the rest was either normal (*n* = 8) or revealed discrete unspecific histological changes (*n* = 39).

Twenty-two patients with a non-specific phenotype had elevated CK levels. In the group of patients with elevated CK, one biopsy was diagnostic, as it showed morphological signs indicative of limb girdle muscular dystrophy (LGMD) (CK level: 306 IU/L). The biopsy result was later genetically confirmed (mutation in the dystrophin gene, Table S2). Later genetic testing revealed a genetic abnormality in two additional patients with non-informative biopsy results (one myotonic dystrophy type 1 in a patient with a CK level of 7300 IU/L and one MYH2 mutation in a patient with a CK level of 1200 IU/L), while genetic testing was negative in four other patients.

In the patients with isolated myalgia and normal CK levels (*n* = 27), one biopsy was diagnostic, as it was indicative of a muscular dystrophy, which could not be further classified. Later genetic testing was negative. In total, four patients were genetically tested, and no mutation was found.

## 4. Discussion

Historically, muscle biopsy has been a key component in the diagnosis of muscle disease. Yet, in an era where the availability of serological, genetic, and auto-antibody testing is continuously increasing, the diagnostic value of muscle biopsy is under debate.

We analyzed the diagnostic value of muscle biopsy in the diagnostic work-up of myopathies from a daily clinical practice perspective. Overall, we found that muscle biopsy in suspected myopathy yielded a completely new diagnosis in 3.7% of the cases and provided a more detailed classification of the clinical diagnosis in an additional 17.3%. The remainder of the biopsies either confirmed the clinically suspected diagnosis or provided non-specific or normal results. The overall concordance between a specific clinical diagnosis and muscle biopsy findings was high, suggesting that specific clinical cues, complemented by relevant antibody testing, mostly led to a reliable diagnosis. In previous literature, the diagnostic yield of muscle biopsy has varied widely depending on the clinical context, patient selection, and therefore, pre-test probability. Study methodologies, e.g., patient selection, laboratory techniques, and outcome parameters, are widely heterogeneous [18]. For adult populations with suspected, clinically undefined, myopathy based on weakness, and/or hyperCKemia, the overall diagnostic yield ranges from 36% [10] to 43% [11], which is higher than the diagnostic yield we reported. This suggests that the added value of muscle biopsies depends on the extent of clinical and laboratory evaluation. Accordingly, our study has shown that the number of diagnostic biopsies strongly depends on the clinical phenotype.

### 4.1. Inflammatory Myopathies

In the majority (75.9%) of patients with inflammatory myopathy, a correct subtype diagnosis could be established based on current clinical criteria, and biopsies were only confirmative. Similar findings were reported in an Austrian multicenter study [19], which reported a histological confirmation rate of 64% in clinically suspected IIM with solid pre-test probability. Compared to studies performed over larger periods of time, nowadays, the diagnostic yield of muscle biopsy might be even lower, as the availability of MSA and MAA testing has gradually increased in recent years [6]. The commercial availability of these antibodies has improved pre-test probability and enhanced clinical phenotyping prior to muscle biopsy, influencing diagnostic algorithms.

Therefore, we suggest muscle biopsies in seronegative patients or those with only weakly positive MSAs, to correctly identify those qualifying for immunosuppression.

### 4.2. Mitochondrial Myopathies

In our cohort of mitochondrial myopathies, a large proportion of patients (29/33) was diagnosed with chronic progressive external ophthalmoplegia (CPEO). This might explain the high proportion of normal biopsies (33.3%), as early CPEO mostly presents without muscle weakness in the limbs, and the deltoid muscle was selected for biopsy according to standard practice. It is important to recognize that pathological changes may be focal, and findings depend on muscle selection. For mitochondrial myopathies, genetic testing of muscle mtDNA plays an important role in the diagnostic process, as mutations in mitochondrial DNA are tissue-specific and might not be detected in blood [20]. In our cohort, genetic testing performed on muscle tissue, frequently histologically normal, revealed genetic alterations in 81.0%. Mitochondrial disease encompasses a group of diseases lacking clear genotype–phenotype correlations. Genetic testing on muscle biopsy is essential to obtain a correct diagnosis to facilitate genetic counselling, including reproductive counselling [20].

### 4.3. Muscular Dystrophies

Hereditary myopathies (e.g., hereditary dystrophies and metabolic myopathies) are relatively rarely diagnosed in adult patients [21]. We included 23 patients with a clinical diagnosis of hereditary muscular dystrophy. We found the percentage of diagnostic muscle biopsies was 17.4%. Nowadays, diagnosis can frequently be obtained using genetic testing. It has been demonstrated that using WES, 47–62% of unsolved cases with unexplained limb girdle weakness could be solved [8,9]. Further molecular studies are still needed to correlate additional causative variants with clinical features to improve the diagnostic yield of WES, as many variants are of unknown significance [22]. As the diagnostic yield of exome sequencing is a lot larger compared to diagnostic yield for muscle biopsy and less invasive, we suggest that genetic testing takes precedence over muscle biopsy early in the diagnostic process. However, for variants of unknown significance or inconclusive WES results, muscle biopsy might still be a useful tool to obtain a diagnosis [23].

### 4.4. Metabolic Myopathies

For metabolic myopathies, 2 out of 3 performed muscle biopsies yielded diagnostic information. Further studies in larger cohorts of patients with metabolic myopathies are required to investigate the diagnostic yield of muscle biopsy and compare it to the diagnostic yield of WES, considering that in some types of metabolic myopathy, e.g., in lipid-storage myopathies, muscle can be structurally normal [24].

### 4.5. Non-Specific Phenotype

Prior studies report that in patients presenting with symptoms suggestive of myopathy, a muscle biopsy is more likely to be diagnostically useful if there is hyperCKemia [25,26]. In patients with clearly defined clinical phenotypes, we found CK levels to be higher in patients with specific biopsy findings (confirmative and diagnostic biopsies) than in patients with non-informative or normal biopsies. In patients with a non-specific phenotype, a diagnostic biopsy was found in only 1 of 22 patients with elevated CK (CK = 306 IU/L, histological pathological features of dystrophinopathy). The high percentage of non-informative biopsies in our cohort of patients with non-specific phenotypes is in line with previous literature suggesting that isolated myalgia or isolated hyperCKemia lacks a high predictive value for positive muscle biopsy [26,27].

To avoid unnecessary investigations in patients with non-specific symptoms, it is suggested that the standard diagnostic approach should only prompt muscle biopsy in the presence of any ‘red flag’ features, such as exertional myalgia, myoglobinuria, second wind-up phenomena, or muscle hypertrophy/atrophy [26].

In some patients without symptoms indicative of a specific myopathy, a normal biopsy can be used to exclude myopathy. However, this should be interpreted with caution, as a normal biopsy can be the result of a sampling error.

## 5. Limitations

This study was conducted over a large time span, in which new diagnostic tools have become available, including the identification of a large number of antibodies found in inflammatory myopathy and advances in and increased access to genetic testing. However, the techniques used in this study for analyzing muscle biopsies have remained largely consistent. The clinical utility of muscle biopsies strongly depends on advances in other diagnostic modalities, and our results should be interpreted in the context of the recent diagnostic advances.

The analytic methods used in this study are not sensitive to all defects, and specific defects might have been overlooked, e.g., isolated defects in complex I, complex III, and complex V, or ragged red fibers in mitochondrial myopathies in histological assessments. Additionally, purely nuclear-coded defects of ATP synthesis, substrate transport, cofactor-metabolism, and/or mitochondrial homeostasis are difficult to detect using standard methods. The handling of muscle biopsies (freezing process) might have negatively influenced the detection rate of defects in complex V.

As inflammatory myopathies with skin manifestations are primarily treated by dermatologists and rheumatologists, such patients might have been underrepresented in our database. Furthermore, at our center, biopsy is often performed in patients with suspected IBM, given that moderate sensitivity of anti-cN1A serology [28] and early IBM can phenotypically overlap with IBM and PM. Histologically, PM-Mito has also been proposed to lie within the spectrum of IBM as a possible early form of the disease [29]. Congenital myasthenic syndromes represent an important differential diagnosis for CPEO, but it was not genetically excluded in our cohort. In congenital myasthenic syndromes, biopsies are most often normal or show only non-specific changes, and mitochondrial changes are not expected [30].

## 6. Conclusions

In many patients, a reliable diagnosis can be established based on the clinical phenotype and, in inflammatory myopathies, antibody testing. Considering the advances in genetic testing, muscle biopsy is of value in selected cases only. Importantly, patients that could still benefit from muscle biopsy are those with weak negative or weak positive MSAs/MAAs that are not in concordance with the clinical phenotype. Additionally, in patients with suspected mitochondrial myopathies, genetic testing on muscle tissue can provide additional information. In patients with suspected muscular dystrophies and negative genetic testing, muscle biopsy could be valuable to confirm the diagnosis.

## Figures and Tables

**Table 1:** Clinical and demographic data, categorized per clinical phenotype.

	Patients, *n* (%)	Age at Biopsy ±SD, y (Range)	Women, *n* (%)	CK Level ± SD, IU/L (Range)
All	162 (100%)	51.7 ± 17.4 (18.3–86.6)	62 (38.0%)	3360.6 ± 19,463.1 (33–200,000) ^†^
Inflammatory Myopathy	54 (33.3%)	65.2 ± 12.5 (33.6–86.6)	22 (40.7%)	8044.6 ± 32,857.4 (66–200,000)
AsyS-/OL myositis	7	59.9 ± 13.0 (33.6–76.1)	4 (57.1%)	875.6 ± 1050.9 (111–2962)
DM	6	65.2 ± 11.6 (51.6–83.0)	4 (66.7%)	1770.2 ± 1429.8 (97–3755)
IBM	23	70.9 ± 9.0 (48.2–84.4)	5 (21.7%)	8480.0 ± 29,328.9 (88–140,000)
IMNM	8	63.0 ± 16.5 (40.2–86.6)	5 (62.5%)	1260.3 ± 2348.4 (69–4966)
No subclassification	10	57.5 ± 11.9 (39.3–74.5)	4 (40.0%)	21,253.7 ± 62,828.2 (66–200,000)
Mitochondrial Myopathy	33 (20.4%)	40.9 ± 16.1 (18.3–69.8)	17 (51.5%)	698.6 ± 2036.1 (33–11,161)
CPEO	29 (14.9%)	38.7 ± 15.6 (18.3–69.8)	15 (51.7%)	744.12 ± 2181.8 (33–11,161) ^‡^
No subclassification	4 (2.5%)	57.4 ± 9.8 (47.6–67.7)	2 (50%)	402.8 ± 523.8 (73–1185)
Muscular Dystrophy	23 (14.2%)	49.3 ± 15.1 (22.7–73.2)	11 (47.8%)	1140.2 ± 1721.8 (97–8237)
LGMD	13 (8.0%)	50.0 ± 14.2 (28.3–68.0)	5 (38.5%)	697.1 ± 664.8 (143–2400)
NEM	1 (0.6%)	73.2	1 (100%)	8237
No subclassification	9 (5.6%)	45.6 ± 15.5 (22.7–65.5)	5 (55.6%)	991.8 ± 922.3 (209–3000)
Metabolic Myopathy No subclassification	3 (1.9%) 3 (1.9%)	46.3 ± 19.3 (24.6–61.4)	0 (0%)	226.0 ± 101.3 (118–319)
Non-specific phenotype	49 (30.2%)	45.6 ± 14.8 (21.2–75.9)	12 (24.5%)	1014.7 ± 2371.3 (38–13,000)
Isolated myalgia	27	51.2 ± 15.2 (23.7–75.9)	6 (50%)	98.2 ± 46.3 (38–178)
Myalgia and hyperCKemia	22	38.6 ± 11.2 (21.2–61.2)	6 (50%)	2102.0 ± 3208.8 (191–13,000)

Abbreviations: AsyS-/OL myositis, antisynthetase syndrome/overlap myositis; CK, creatine kinase; CPEO, chronic progressive external ophthalmoplegia; DM, dermatomyositis; LGMD, limb girdle muscular dystrophy; IBM, Inclusion Body Myositis; IMNM, immune-mediated necrotizing myositis; NEM, neck extensor myopathy; PM, Polymyositis; SD, standard deviation;. ^†^ CK-level available in 159 patients. ^‡^ CK-level missing in 3 patients.

**Table 2:** Diagnostic yield of muscle biopsies among different clinical phenotypes.

Clinical Diagnosis	Diagnostic Biopsy	Confirmative Biopsy	Non-Informative Biopsy
Total (*n* = 162)	34 (21.0%)	62 (38.3%)	65 (40.1%)
Inflammatory myopathy (*n* = 54)	8 (14.8%)	41 (75.9%)	5 (9.3%)
Mitochondrial myopathy (*n* = 33)	18 (54.5%)	4 (12.1%)	11 (33.3%)
Muscular dystrophy (*n* = 23)	4 (17.4%)	16 (69.6%)	3 (13.0%)
Metabolic myopathy (*n* = 3)	2 (66.7%)	1 (33.3%)	0 (0.0%)
Non-specific phenotype (*n* = 49)	2 (4.1%)	0 (0.0%)	47 (95.9%) ^‡^

^‡^ In patients with a non-specific phenotype (myalgia and/or hyperCKemia without further symptoms), 8 patients had normal biopsies, while 39 patients had discrete unspecific histological alterations, such as type I fiber predominance, sparse internalized cell nuclei, isolated atrophic muscle fibers, or slight variations of fiber caliber.

**Table 3:** Antibody profiles of patients with a clinical diagnosis of myositis.

Clinical Diagnosis	Antibodies	Number of Patients
IBM (*n* = 23)	MUP4/cN1A	3
	Seronegative	10
	Not tested	10
DM (*n* = 6)	Jo-1	1
	NXP-2	1
	NXP-2 + TIF1-γ	1
	Seronegative	3
	Not tested	0
IMNM (*n* = 8)	HMG Co-A	6
	SRP	2
	Seronegative	0
	Not tested	0
Overlap syndrome (*n* = 7)	Jo-1	1
	PM-Scl	2
	Ro52	1
	EJ	1
	Ro60	1
	ANA	1
	Not tested	0
Inflammatory myopathy, unspecified (*n* = 10)	SRP	2
	Ro-52	1
	Jo-1	1
	Seronegative	3
	Not tested	3

Abbreviations: ANA, Antinuclear Antibodies; DM, dermatomyositis; EJ, Glycyl-tRNA Synthetase Antibody; HMG Co-A, 3-Hydroxy-3-Methylglutaryl-Coenzyme A Reductase; IBM, Inclusion Body Myositis; IMNM, Immune-Mediated Necrotizing Myopathy; Jo-1, Histidyl-tRNA Synthetase; MUP4/cN1A, Cytosolic 5′-Nucleotidase 1A; NXP-2, Nuclear Matrix Protein 2; TIF1-γ, Transcription Intermediary Factor 1-Gamma; PM-Scl, Polymyositis–Scleroderma; Ro52, TRIM21; Ro60, TROVE2; SRP, Signal Recognition Particle.

## Data Availability

The datasets obtained and/or analyzed during the current study are available from the corresponding author on reasonable request as the data are not publicly available due to privacy restrictions.

## Supplementary Materials

The following supporting information can be downloaded at https://www.mdpi.com/article/10.3390/diagnostics15243102/s1, Table S1: Immunoblot findings in muscle biopsy tissue of patients with a clinical diagnosis of muscular dystrophy; Table S2: Biopsy results in patients with hyperCKemia and/or myalgia, based on clinical characteristics; Table S3: Overview of diagnostic procedures on muscle for the work-up of myopathies.
