# Supplementary material for: Diagnostic Value of Muscle Biopsy for the Evaluation of Adult Myopathy in Daily Clinical Practice
Source: Diagnostics (Basel). 2025 Dec 6;15(24):3102. doi: 10.3390/diagnostics15243102 (PMC12732179; doi:10.3390/diagnostics15243102)
Supplement: Supplementary file 1 [file diagnostics-15-03102-s001.zip › diagnostics-3997272-supplementary.pdf]

Supplementary Materials:

Supplementary Table S1: Immunoblot findings in muscle biopsy tissue of patients with a clinical diagnosis of muscular dystrophy

Patients	Biopsy diagnosis	Immunoblot result
**Patient 1.**	LGMD, unspecified	α-Dystroglycan
**Patient 2.**	LAMP-2 Danon disease	LAMP-2 deficiency
**Patient 3.**	LGMD, type 2A	Calpain-3

Abbreviations: LAMP-2, lysosome-associated membrane protein 2; LGMD, Limb girdle muscular dystrophy.

Genetic testing was not performed in all 3 patients.

Supplementary Table S2. Biopsy results in patients with hyperCKemia and/or myalgia, based on clinical characteristics

Biopsy diagnosis	Number of patients (%)
**Myalgia and hyperCKemia**	22 (100%)
Non-informative	21 (95.5%)
LGMD (genetically confirmed dystrophinopathy)	1 (4.5%)
**Isolated myalgia (normal CK)**	27 (100%)
Non-informative	26 (96.3%)
Muscular dystrophy, no subclassification	1 (3.7%)

Abbreviations: CK, creatine kinase; LGMD, limb girdle muscular dystrophy.

Supplementary Table S3: Overview of diagnostic procedures on muscle for the work-up of myopathies

Inflammatory myopathies	Immunostaining for CD3, CD8, CD20, CD68, MHC class I, C5b-9<br>IBM associated proteins TDP43, SM31, p62 and ubiquitin<br>Electron microscopy for tubulo-filamentous inclusions
Muscle dystrophies	Immunostaining for Dystrophin and Dystrophin-associated proteins<br>Western blot analysis of Dystrophin N-, C-terminus and rod domain, Calpain-3, Caveolin-3, Lamin A/C, Dysferlin, Merosin, Telethonin, and α-, *β*-, γ-Dystroglycans, Desmin, αβ-Crystallin, Myotilin

Metabolic myopathies	PAS, Oil Red O, Sudan black B, Myo-phosphorylase, Phosphofructokinase, Myo-adenylate deaminase
Mitochondrial myopathies	NADH, SDH, COX<br>Electron microscopy for mitochondria<br>Biochemical analysis of oxidative enzymes,<br>Genetic analysis of mitochondrial DNA

Abbreviations: CD3, Cluster of Differentiation 3; CD8, Cluster of Differentiation 8; CD20, Cluster of Differentiation 20; CD68, Cluster of Differentiation 68; C5b-9, Complement Membrane Attack Complex; COX, Cytochrome c Oxidase; EM, Electron Microscopy; IBM, Inclusion Body Myositis; MHC class I, Major Histocompatibility Complex Class I; Myo-AD, Myo-adenylate Deaminase; Myo-PFK, Muscle Phosphofructokinase; Myo-PHO, Muscle Phosphorylase; NADH, Nicotinamide Adenine Dinucleotide; PAS, Periodic Acid–Schiff Stain; p62, Sequestosome-1 (SQSTM1); SDH, Succinate Dehydrogenase; SM31, Stress Marker 31; TDP-43, TAR DNA-Binding Protein 43; α-DG, Alpha-Dystroglycan; β-DG, Beta-Dystroglycan; γ-DG, Gamma-Dystroglycan
